# The conserved multi-functional nuclear protein *dss-1/Sem1* is required for C9orf72-associated ALS/FTD dipeptide toxicity

**DOI:** 10.17912/micropub.biology.000262

**Published:** 2020-06-02

**Authors:** Noah Puleo, Todd Lamitina

**Affiliations:** 1 University of Pittsburgh, Depts. of Pediatrics and Cell Biology

**Figure 1:**
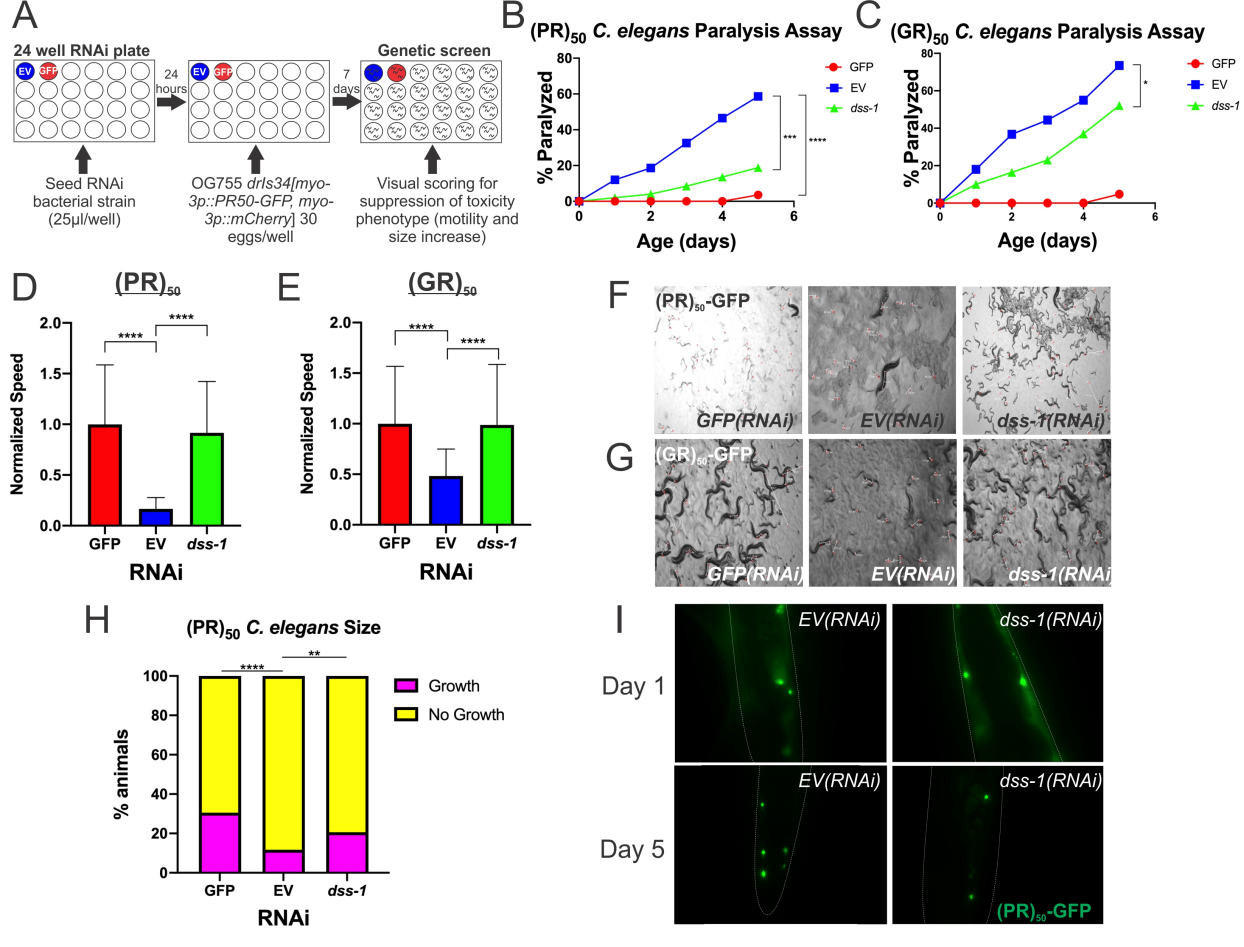
*dss-1* is required for the toxicity of C9orf72-DPRs Proline-Arginine (PR) and Glycine-Arginine (GR) in *C. elegans* and acts downstream of DPR nuclear localization. (A) NGM 24 wells were seeded with 25 µL/well of an ORFeome-specific collection of RNAi bacterial strains targeting 1,691 genes. After 24 hours, gravid *C. elegans* expressing the *drIs34* transgene (Rudich *et al.* 2017) were bleached and their eggs were seeded into the 24 wells. Seven days after incubation at 20°C, animals were screened for suppression of growth arrest and paralysis as compared to *EV(RNAi)* control. (B) Paralysis assay using (PR)_50_ *C. elegans* straincomparing *dss-1(RNAi)* to *GFP(RNAi)* and *(EV)RNAi*. N = 50 animals for each genotype. ****P < 0.0001, ***P < 0.0005 (Log-rank test with Bonferroni adjusted P-value). Experimental details found in Rudich *et al.*, 2020. (C) Paralysis assay using (GR)_50_ *C. elegans* strain comparing the indicated genotypes. N = 50 animals for each genotype. *P < 0.05 (Log-rank test with Bonferroni adjusted P-value). (D) Video speed analysis using (PR)_50_ *C. elegans* strain comparing the indicated genotypes. N = 30-40 animals for each genotype. ****P < 0.0001 (one-way ANOVA with Bonferroni correction). (E) Video speed analysis using (GR)_50_ *C. elegans* strain comparing the indicated genotypes. N = 40-48 animals for each genotype. ****P < 0.0001 (one-way ANOVA with Bonferroni correction). (F-G) Images captured after finding the distances for (F) (PR)_50_ *C. elegans* strain and (G) (GR)_50_ *C. elegans* strain in relation to the video speed analysis. Each line in the image represents the movement of one *C. elegans* over time. For further details, see Rudich *et al.* 2020. (H) Worm length (time-of-flight (TOF)) of individual (PR)_50_ *C. elegans* recorded via COPAS sorting. “Growth” vs “No Growth” threshold was empirically determined at a TOF of 300. TOFs above (pink) and below (yellow) this threshold was recorded for each RNAi and reported as a percentage of the total population. *GFP(RNAi)*: N = 262, *EV(RNAi)*: N = 257, *dss-1(RNAi)*: N = 267. ****P < 0.0001, **P = 0.0063 (Fisher’s exact test). (I) Images taken fromfluorescent microscopy of (PR)_50_-GFP expressed in *C. elegans* muscle at day 1 and day 5 after transfer from *GFP(RNAi)* to *EV(RNAi)* and *dss-1(RNAi)*.

## Description

Neurodegenerative diseases caused by short expansive repeats like the (CAG) in Huntington’s disease (Orr 2012) or the (GGGGCC) repeat in C9orf72-associated Amyotrophic lateral sclerosis (ALS)/Frontotemporal dementia (FTD) (DeJesus-Hernandez *et al.* 2011) undergo an unusual type of translation called repeat associated non-AUG-dependent (RAN) translation (Cleary and Ranum 2014). Interestingly, RAN translation occurs without an AUG start codon (Cleary and Ranum 2014). This allows for the (GGGGCC) repeat mutation to be translated, even though it is located in the intron between exon 1 and exon 2 of the C9orf72 gene, which would normally be spliced out and degraded (DeJesus-Hernandez *et al.* 2011). Translation of the repeat occurs in all 3 reading frames, leading to the production of three distinct dipeptide repeat proteins (DPRs). RAN translation begins within the (GGGGCC) repeat, but the exact translation initiation site remains unclear. However, RAN translation does not stop at the end of the repeat and will continue to translate the intronic sequence until it reaches a stop codon. This means that each of the distinct DPRs will be fused to peptides encoded in the downstream intron sequence. Because the DPRs are derived from intron sequence that is spliced out of the mature C9orf72 mRNA, none of these intron-derived DPR fusion peptides are incorporated into the ‘normal’ C9orf72 protein. While it is known that the DPR fusion peptides are made in patients, the precise sequences of the DPR fusion peptides that they produce is not currently known. Therefore, questions about where precisely RAN translation initiates, how many repeats are produced, and whether the number of repeats produced are uniform or heterogenous remain important but unresolved questions.

There is also a C9orf72 antisense transcript, which contains the complementary repeat sequence (GGCCCC). This antisense transcript also undergoes RAN translation to produce another three DPRs (Zu *et al.* 2013). Therefore, a single DNA repeat expansion in one gene gives rise to six distinct DPRs. These DPRs form p62 positive/pTDP-43 negative inclusions that are distinct hallmarks of C9orf72-associated ALS/FTD (Cleary and Ranum 2014). Our laboratory as well as others have shown that two of these DPRs, proline-arginine (PR) and glycine-arginine (GR) are highly toxic (Kwon *et al.* 2014; Wen *et al.* 2014; Rudich *et al.* 2017), however the mechanisms of toxicity are poorly defined.

In order to study the mechanisms that cause C9orf72-associated ALS/FTD PR and GR toxicity, we utilized the *Caenorhabditis elegans* model system. With short lifespans (3-4 weeks), a conserved neuromuscular system, and a genome that encodes ~20,000 genes with many conserved human homologs, the *C. elegans* model system is highly relevant for the study of aging and age-related diseases like ALS (Olsen *et al.* 2006). To study how PR and GR cause toxicity in *C. elegans*, we created animals expressing codon-optimized (PR)_50_-GFP and (GR)_50_-GFP (Rudich *et al.* 2017). With this approach, we are able to observe the effects of a single DPR at a time, without additional contributions from either the loss of the C9orf72 gene expression, introduction of the G_4_C_2_ repeat containing RNA, or the other five RAN translated DPRs. Therefore, this is a pure DPR model. Our laboratory has previously shown (PR)_50_ and (GR)_50_ to be toxic by causing a decrease in motility (paralysis) and arrested growth, when expressed in muscle (Rudich *et al.* 2017). Nuclear localization of these two DPRs was also discovered to be necessary and sufficient for toxicity in *C. elegan*s (Rudich *et al.* 2017).

Using a transgenic *C. elegan*s (PR)_50_ line, we screened RNAi feeding clones targeting 1,691 genes that are unique to the ORFeome RNAi library and not found in the MRC/Ahringer RNAi library. *GFP(RNAi)* and *EV(RNAi)* functioned as positive and negative controls respectively (Figure 1A). After two rescreens of the initial hits from the 1,691 genes screened, we identified two RNAi knockdowns that were able to suppress the loss of motility and arrest in growth caused by (PR)_50_. These two hits were subsequently screened six additional times, all of which suppressed (PR)_50_ growth arrest and paralysis. Both RNAi clones were sequenced and the sequences were aligned to the *C. elegans* genome using BLAST to identify the affected genes**.** The two genes uncovered in the screen were *lin-54* and *dss-1*. In most assays, *dss-1(RNAi)* had a much stronger toxicity suppression phenotype than *lin-54(RNAi)* and rescued motility back to near wildtype levels. Because the *dss-1(RNAi)* phenotype was robust, additional mechanistic studies were performed to understand how *dss-1* contributes to DPR toxicity.

One possible mechanism for the observed suppression was a decrease in the activity of the promoter (*myo-3p*) controlling (PR)_50_ gene expression. In *C. elegans* RNAi screens utilizing transgene-based phenotypes (such as the transgenic PR expression utilized here), one known class of suppressors include genes whose inhibition globally suppresses transgene expression (Fischer *et al.* 2013). Large object flow cytometry (COPAS sorting) was used to quantitatively measure the expression level of a fluorescent reporter (Red fluorescent protein, RFP) derived from the PR transgene which utilizes the same *myo-3* promoter (Rudich *et al.* 2017). Normalized RFP levels (RFP/TOF) were not significantly different between *EV(RNAi)* and *dss-1(RNAi)* (*(EV(RNAi)* – .04615 +/- .03731; N = 30; *dss-1(RNAi)* – .05432 +/- .08057; N=55; ANOVA with post-hoc test, p > 0.9999). Therefore, *dss-1(RNAi)* does not act via transgene suppression.

When (PR)_50_ is expressed post-developmentally in adult *C. elegans*, it causes an age-dependent paralysis phenotype. *GFP(RNAi)* fully suppresses age-dependent paralysis and restores motility, strongly suggesting that this phenotype is due to the expression of the (PR)_50_ protein. Using this paralysis assay, we examined the effect that *dss-1(RNAi)* had on age-dependent paralysis of *C. elegans* expressing (PR)_50_. Two independent paralysis assays showed that animals on *dss-1(RNAi)* exhibited reduced paralysis compared to the negative control *EV(RNAi)* (Figure 1B). In addition to these phenotypes, the other toxic DPR made via the C9orf72 repeat expansion mutation, (GR)_50_, showed similar results (Figure 1C). This decrease in *C. elegans* age-dependent paralysis following *dss-1(RNAi)* shows that *dss-1* normally functions to facilitate (PR)_50_ and (GR)_50_ induced age-dependent paralysis.

When (PR)_50_ is expressed developmentally in muscle, it causes strong larval paralysis. Larval paralysis is completely suppressed by *GFP(RNAi)*, strongly suggesting that this phenotype is due to the expression of the (PR)_50_-GFP protein. In order to examine the effect that (PR)_50_ has on the developmental paralysis phenotype, we developed a novel method called video speed analysis (VSA) (Rudich *et al.* 2020). Two independentVSA trials showed increased motility in (PR)_50_ expressing animals on *dss-1(RNAi)* compared to *EV(RNAi)* (Figure 1D, F). (GR)_50_, showed similar results (Figure 1E, G). These results display that *dss-1* normally functions to help facilitate PR and GR induced developmental paralysis.

When (PR)_50_ is expressed developmentally in muscle, it causes an arrest in the growth of *C. elegans* along with a decrease in motility. This results in the accumulation of short larvae and a depletion of longer adult worms. Feeding animals *GFP(RNAi)* completely suppresses this growth arrest, strongly suggesting this phenotype is due to the (PR)_50_ protein. In order to quantify the effect of (PR)_50_ +/- *dss-1(RNAi)* on *C. elegans* size, *C. elegans* length was measured via COPAS biosorting on *GFP(RNAi)*, *EV(RNAi)*, and *dss-1(RNAi)* (Figure 1H). Setup of *C. elegans* for sorting followed the same methodology and time frame as the RNAi genetic screen. ‘Growth’ or ‘No Growth’ thresholds were empirically determined based on the sizes observed in the positive (*GFP(RNAi)*) and negative (*EV(RNAi)*) controls. Worms above this threshold were labeled as ‘Growth’ and worms below this threshold were labeled as ‘No Growth.’ Results from this experiment showed that *C. elegans* increased in size on *dss-1(RNAi)* compared to *EV(RNAi)*, showing that *dss-1(RNAi)* suppressed the growth arrest caused by (PR)_50_ (Figure 1H). This result shows that *dss-1* normally functions to help facilitate (PR)_50_-induced suppression of growth.

As we previously discovered in our laboratory, nuclear localization is required for (PR)_50_ and (GR)_50_ toxicity in *C. elegans* (Rudich *et al.* 2017). Therefore, *dss-1(RNAi)* could suppress (PR)_50_ toxicity either because it prevents (PR)_50_ localization to the nucleus or because it inhibits relevant toxicity pathways downstream of nuclear (PR)_50_. To distinguish between these possibilities, we used fluorescence microscopy to determine if (PR)_50_-GFP localization is altered by *dss-1(RNAi)* (Figure 1I). In *dss-1(RNAi)* animals, PR nuclear localization was unaffected. Therefore, *dss-1* is not required for PR nuclear localization but rather facilitates the (PR)_50 _toxicity mechanism(s). The possible mechanisms through which *dss-1* functions in this pathway to cause toxicity are unclear and will be a point of future research.

Through these results, we have shown that *dss-1* is required for C9orf72-associated ALS/FTD. *dss-1* (Deleted in Split hand/Split foot protein 1) is a small conserved (human ortholog *Sem1*) multi-functional nuclear protein involved in multiple different cellular processes (Pispa *et al.* 2008). *dss-1* is part of the 26S proteasome, helps assemble the 19S subunits of the 26S proteasome, and binds ubiquitin (Kragelund *et al.* 2016). While it is still unclear what the ubiquitin binding role(s) of *dss-1* are in the 26S proteasome, it is hypothesized that it may act as a ubiquitin receptor domain within the proteasome (Paraskevopoulos *et al.* 2014). In addition, *dss-1* is also part of the Sac3-Thp1, or TREX-2, complex which is involved in mRNA export as well as the Csn12-Thp3 complex which is involved in RNA splicing in the nucleus (Wilmes *et al.* 2008). Both the proteasome and mRNA export/splicing have been previously linked to C9orf72-associated DPR toxicity (Boeynaems *et al.* 2017, Gupta *et al.* 2017, Kramer *et al.* 2018, Kwon *et al.* 2014, Lee *et al.* 2016).

*dss-1* is characterized as an intrinsically disordered protein due to its lack of defined structure and its ability to have multiple protein conformations (Kragelund *et al.* 2016). Interestingly, it has been shown that many intrinsically disordered proteins promote liquid-liquid phase separation (LLPS), an important organizing mechanism for intracellular compartmentalization of some proteins (Alberti 2017). The disruption of LLPS has been identified in multiple neuropathologies, including C9orf72-associated ALS/FTD (Boeynaems *et al.* 2017). Toxic DPRs PR and GR have been shown to be able to phase separate, which can disrupt LLPS of nucleoli, the nuclear pore complex and stress granules (Lee *et al.* 2016; Boeynaems *et al.* 2017). These stress granules contain important RNA binding proteins like TDP-43 and FUS (Boeynaems *et al.* 2017). PR and GR have been shown to disrupt these stress granules by causing liquid-to-solid maturation (Boeynaems *et al.* 2017). This liquid-to-solid formation is a hallmark of neurodegenerative pathogenesis and triggers an acceleration of ALS phenotypes (Patel *et al.* 2015). Together, these observations suggest that *dss-1* may play an important role in LLPS pathology that influences ALS/FTD toxicity.

A recent study shows that the proteasome, including the 26S regulatory subunit with which *dss-1* interacts, also undergoes ubiquitin-dependent LLPS in the nucleus (Yasuda *et al.* 2020), suggesting a plausible connection among *dss-1*, C9orf72-associated PR and GR liquid-liquid phase separation (LLPS), and the proteasome. These inter-connections include: PR and GR mediated inhibition of ubiquinated substrates (Gupta *et al.* 2017); CRISPR-Cas9 screens in K562 cells and primary mouse neurons showing proteasome subunits as genetic modifiers of PR and GR (Kramer *et al.* 2018); and C9orf72-associated impairment of TDP-43 degradation by the proteasome (Lee *et al.* 2019). However, *dss-1* may offer a new link in regard to C9orf72-associated ALS/FTD and the proteasome. One potential hypothesis is that *dss-1* helps facilitate LLPS disruption of the proteasome by C9orf72-associated toxic DPRs PR and GR. Since ubiquitinated substrates are required for liquid droplet formation of the proteasome (Yasuda *et al.* 2020), the role of *dss-1* as a ubiquitin receptor in the proteasome (Paraskevopoulos *et al.* 2014) may help in the formation of liquid droplets. Loss of *dss-1* could be protective because there is less LLPS of the proteasome due to a lack of recruitment of ubiquinated substrates. Therefore, with a reduction in LLPS, the toxic DPRs can no longer cause liquid-to-solid maturation which has been shown to be harmful (Patel *et al.* 2015). Future experimentation testing this hypothesis, along with investigation into the other known functions of *dss-1* in mRNA nuclear export and mRNA splicing, may lead to insights into a conserved mechanism by which *dss-1* is required for PR and GR toxicity.

In conclusion, this study highlights *dss-1* as a key component of the C9orf72-associated ALS/FTD genetic pathway and suggests *dss-1* as a potential therapeutic target for treatment of ALS/FTD. This study has shown that *dss-1* inhibition is able to rescue developmental and post-developmental motility as well as developmental size defects in *C. elegan*s expressing the toxic (PR)_50_ and (GR)_50_ dipeptide repeat proteins. Future studies in mice and human cell culture are needed to evaluate the potential efficacy of *dss-1* antagonists as a therapeutic option for treating C9orf72-associated ALS/FTD patients.

## Reagents

*C. elegans* Strains: OG755 +/+; *drIs34* [*myo-3p::(PR)_50_-GFP*; *myo-3p::mCherry*] X and OG736 +/+; *drIs28* [*myo-3p::(GR)_50_-GFP*; *myo3p::mCherry*] (integration site unmapped); *C. elegans* ORFeome RNAi library (Dharmacon/Horizon Discovery, Cambridge, UK), *dss-1* RNAi clone ORF ID – Y119D3B.15
